# Bending Response of 3D-Printed Titanium Alloy Sandwich Panels with Corrugated Channel Cores

**DOI:** 10.3390/ma14030556

**Published:** 2021-01-24

**Authors:** Zhenyu Zhao, Jianwei Ren, Shaofeng Du, Xin Wang, Zihan Wei, Qiancheng Zhang, Yilai Zhou, Zhikun Yang, Tian Jian Lu

**Affiliations:** 1 State Key Laboratory of Mechanics and Control of Mechanical Structures, Nanjing University of Aeronautics and Astronautics, Nanjing 210016, China; zhenyu_zhao@nuaa.edu.cn (Z.Z.); jeawren0621@nuaa.edu.cn (J.R.); yilaizhou@nuaa.edu.cn (Y.Z.); 2 MIIT Key Laboratory of Multi-Functional Lightweight Materials and Structures, Nanjing University of Aeronautics and Astronautics, Nanjing 210016, China; wxtj_9449@stu.xjtu.edu.cn (X.W.); wzh123@stu.xjtu.edu.cn (Z.W.); zqc111999@xjtu.edu.cn (Q.Z.); 3 State Key Laboratory of Smart Manufacturing for Special Vehicles and Transmission System, Baotou 014030, China; yzkwh@163.com; 4 State Key Laboratory for Strength and Vibration of Mechanical Structures, Xi’an Jiaotong University, Xi’an 710049, China

**Keywords:** sandwich panels with corrugated channel core, 3D-printed sandwich, bending response, mechanism maps, geometrical optimization

## Abstract

Ultralight sandwich constructions with corrugated channel cores (i.e., periodic fluid-through wavy passages) are envisioned to possess multifunctional attributes: simultaneous load-carrying and heat dissipation via active cooling. Titanium alloy (Ti-6Al-4V) corrugated-channel-cored sandwich panels (3CSPs) with thin face sheets and core webs were fabricated via the technique of selective laser melting (SLM) for enhanced shear resistance relative to other fabrication processes such as vacuum brazing. Four-point bending responses of as-fabricated 3CSP specimens, including bending resistance and initial collapse modes, were experimentally measured. The bending characteristics of the 3CSP structure were further explored using a combined approach of analytical modeling and numerical simulation based on the method of finite elements (FE). Both the analytical and numerical predictions were validated against experimental measurements. Collapse mechanism maps of the 3CSP structure were subsequently constructed using the analytical model, with four collapse modes considered (face-sheet yielding, face-sheet buckling, core yielding, and core buckling), which were used to evaluate how its structural geometry affects its collapse initiation mode.

## 1. Introduction

Increasing demand for integration and miniaturization of load-sustaining mechanical structures calls for novel lightweight materials. Various methodologies have been developed to construct high-performance lightweight structures, e.g., selection of lightweight base materials [1], structural/topological optimization based on machine learning [2,3], bio-inspired design such as hierarchical [4] and graded [5] strategies and their combination, and so on. The essence of the above method is to remove redundant mass by optimizing the structure topology and size. Nowadays, however, a load-bearing mechanical structure is often required to possess additional attributes, e.g., energy absorption, sound attenuation, heat/mass transfer, energy storage, and so on [6,7,8,9]. Design and construction of lightweight multifunctional structures have thus become a focal point in a wide range of applications. 

High-porosity cellular metals with either open or closed cells have emerged as attractive multifunctional materials [10]. Particularly, for applications requiring simultaneous load bearing and active cooling, sandwich constructions with three-dimensional (3D) lattice trusses or two-dimensional (2D) prismatic cores have been extensively exploited [11]. For instance, an all-metallic corrugated sandwich panel is a typical multifunctional structure: in addition to heat dissipation [1,12], it can effectively absorb sound if micro-perforations are introduced to both its face sheets and core webs [13,14]; it can also provide effective projectile penetration resistance if ceramic or concrete prisms are inserted to the interstices of its corrugated core [15,16,17]. Nonetheless, in terms of specific stiffness and specific strength, a corrugated sandwich construction is inferior to other competing structures such as honeycomb-cored sandwiches, especially when subjected to bending and shearing [18,19]. More recently, to maintain the flow-through topology of the corrugated core and improve its load-carrying capability, a novel corrugated channel core (i.e., periodic fluid-through wavy passages) was envisioned for multifunctional sandwich constructions [20]. It was demonstrated, both theoretically and experimentally, that all metallic, corrugated-channel-cored sandwich panels (3CSPs) exhibit not only significantly enhanced convective heat transfer rate, but also superior mechanical performance relative to sandwich panels with parallel plate channels widely used for active cooling. Further, when subjected to the loading of out-of-plane compression, the proposed corrugated channel core exhibits superiority, particularly in the low-density regime, in comparison with competing sandwich core topologies (e.g., square honeycombs, hollow pyramidal trusses, and octet trusses) [20]. Upon inserting PMI foam blocks into the interstices of the corrugated channel core made of a purpose-made fiber-reinforced composite, it was also shown theoretically and experimentally that the resulting hybrid-cored sandwich panel (with composite face sheets) not only exhibits superior specific stiffness/strength but also is endowed with a new attribute, i.e., microwave absorption/transmission [21].

With ever increasing demand for lightweight multifunctional structures, we envision that the novel all-metallic 3CSP construction proposed in our previous study holds great potential for a wide variety of engineering applications, targeting in particular simultaneous load-bearing and heat dissipation via active cooling at ultra-lightweight. To this end, processing methods suitable for fabricating high-quality 3CSP structures need to be exploited, especially when high performance metals such as titanium alloys are used as the parent material. Further, in addition to systematically studying the heat transfer performance of an as-fabricated 3CSP specimen, its mechanical performance also needs to be investigated comprehensively. Furthermore, analytical and numerical models need to be established to explore in detail the physical mechanisms that underlie the experimentally observed mechanical responses.

The objectives of this study were therefore three-fold. Firstly, the method of selective laser melting (SLM) was employed to fabricate Ti-6Al-4V 3CSP sandwich panels for the first time. Previously, these panels were fabricated using a four-step procedure: (a) folding of corrugated sheet; (b) fabrication of corrugated web; (c) assembling of sandwich panel; and (d) vacuum brazing [20,22]. Under bending and/or shear loading, 3CSP specimens thus fabricated with relatively thin face sheets and core web plates will not reach the pre-designated strength, because it is difficult to weld the thin face sheets with the thin core webs, thus yielding inferior shear strength. The SLM technique adopted in this study can squarely address this deficiency, because the pressing demand for high performance 3CSPs as ultra-lightweight multifunctional structures in fields such as aerospace might compromise the relatively high cost of SLM. Secondly, in addition to the out-of-plane uniform compression considered in [20], the performances of as-fabricated Ti-6Al-4V 3CSP sandwich panels subjected to four-point bending were systematically characterized using a combined experimental, analytical, and numerical approach. Thirdly, the analytical model, validated against experimental measurements, was employed to construct collapse mechanism maps, thus providing an effective strategy for designing 3CSP structures with optimal bending responses. Eventually, upon performing the foregoing tasks, we demonstrated that four competing collapse initiation modes governed the failure processes of the Ti-6Al-4V 3CSP structure as its geometry was varied, and the collapse mechanism maps constructed using the developed analytical model provides an effective strategy for designing 3CSP structures with optimal mechanical responses.

The results of this study demonstrate that the proposed 3CSP structure indeed holds great potential in multifunctional applications demanding simultaneous load-bearing and heat dissipation at ultra-lightweight.

## 2. Methodologies

### 2.1. Fabrication of 3CSP Specimens

#### 2.1.1. Topology of 3CSP Specimen

**Figure 1:**
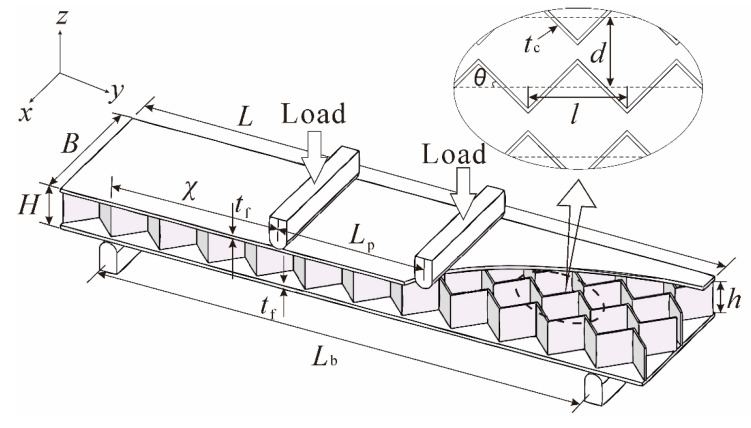
Schematic of sandwich panel with corrugated channel core subjected to four-point bending.

As depicted schematically in Figure 1, the all-metallic sandwich panel investigated in the current study is composed of two face sheets of equal thicknesses (*t*_f_) and a corrugated channel core with a thickness *t*_c_ and a height *h*. Let *L*, *B*, and *H* represent the length, width, and height of the sandwich panel, respectively. The idealized core profile is also shown in Figure 1, which presents a triangular corrugation core with a wavelength *l* and an inclination angle *θ*. The equivalent neutral surfaces of the webs are parallel to each other, with spacing *d*. The face sheets and the core are made of the same metallic material (i.e., Ti-6Al-4V). For the present four-point bending test, as detailed in Section 2.2, the distance between the two indenters (*L*_p_) is fixed at 34 mm, while the span between the two bearings (*L*_b_) is fixed at 119 mm. The diameter of the indenter/bearing is 10 mm. The characteristic length of the specimen is thus given by (Figure 1): $\chi =(L_{b}-L_{p})/2$. The weight per unit area *W* denotes the weight of a structure per unit surface area [18]. For the present 3CSP, it is given by $W=\frac{M_{f}+M_{c}}{a}=\;2t_{f}\rho_{s}+\frac{t_{c}h}{d\cos\theta }\rho_{s}$ where $M_{f}=2t_{f}LB\rho_{s}$ and $M_{c}=\frac{Lt_{c}hB}{d\cos\theta }\rho_{s}$ are the weight of the face sheets and the core, respectively; a=LB is the surface area of the 3CSP; and $\rho_{s}$ is the density of its parent material. Additionally, to facilitate the quantification of structural mass, a dimensionless metric $\psi =W/\rho_{s}\chi$ is defined in this study. Subject to the limits of fabrication precision and specimen dimensions, the specimens are envisioned to vary systemically in geometry, as listed in Table 1. It should be mentioned that, in Figure 1, the face sheets and the core webs are not welded together as done in our previous study [20]. Rather, the two formed an integral part as 3D printing is used in the current study to construct the 3CSP structure from scratch. Indeed, no interaction layer is present between the core web and the face sheet in the as-fabricated Ti-6Al-4V 3CSP specimen.

#### 2.1.2. Fabrication Methodology

**Figure 2:**
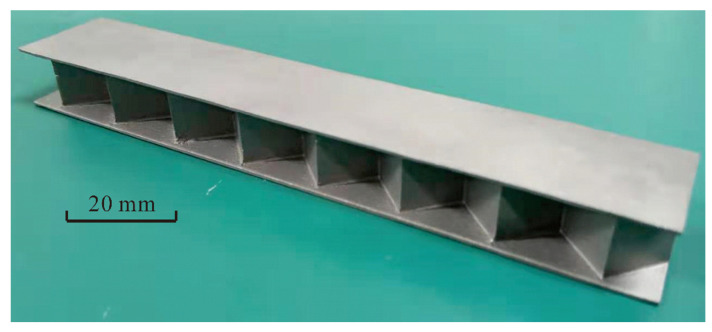
Ti-6Al-4V alloy 3CSP fabricated with the SLM methodology.

**Figure 3:**
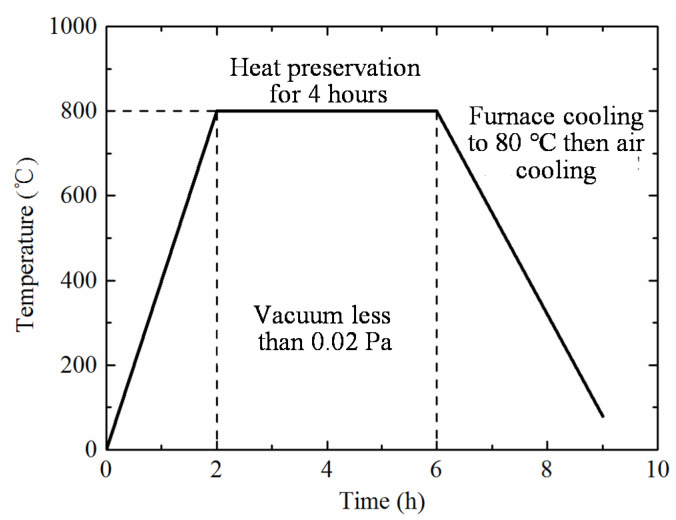
Thermal treatment procedure for Ti-6Al-4V alloy 3CSP specimen.

The method of selective laser melting (SLM) enables manufacturing metal structures via additive printing layer by layer [23,24]. Because it completely melts a powder material, the SLM methodology allows for a density of approximately 100% and assures series-identical properties for fabricated specimens [25]. Due to the relatively complex geometry of the proposed 3CSP (Figure 1), all specimens in this study (Table 1) were fabricated via SLM. Figure 2 presents a typical Ti-6Al-4V sandwich panel thus fabricated using EOS M290 (EOS Ltd. Germany). The device is equipped with an IPG fiber laser having a power of 400 W and an equivalent speckle diameter of 100 µm. The IPG fiber laser outputs a laser in continuous wave (CW) mode. A checkerboard scanning strategy was adopted during the printing process, and the laser heat source was moved from the top to the bottom while also moving from left to right. The corresponding processing parameters are presented in Table 2, while geometries of as-fabricated specimens are listed Table 3, together with measured deviations from those envisioned (Table 1). Subsequently, thermal treatment of the fabricated specimens was carried out to minimize stress localization (Figure 3), which is composed of three phases: a linear heating period from 0 to 800 °C, with a heating velocity of 400 °C per hour; subsequent maintaining period at a constant temperature of 800 °C for 4 h; and a furnace cooling treatment of 240 °C per hour, from furnace temperature (800 °C) to 80 °C. Lastly, air cooling proceeded until the specimen temperature reaches ambient temperature. Otherwise, a furnace vacuum condition with a base pressure less than 0.02 Pa was used during the entire thermal treatment.

**Figure 4:**
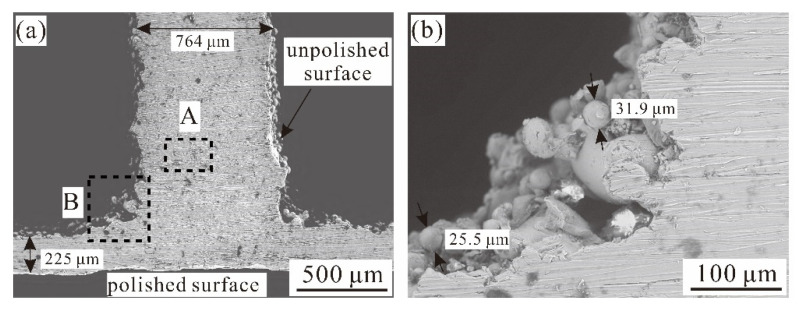
As-fabricated Ti-6Al-4V alloy 3CSP specimen: (**a**) SEM observation with low magnification view; and (**b**) detail of joint region in (**a**) marked by dashed line A.

Figure 4 displays the scanning electron microscope (SEM; Hitachi TM-4000, Hitachi High-Tech Corporation, Tokyo, Japan) images for selected connection region between the face sheet and the corrugated channel core. Built upon these SEM observations, relative size offsets between the as-fabricated specimens and the envisioned specimens were measured, as summarized in Table 3. The largest discrepancy (12.5%) occurs on the face sheet of Specimen VII. In general, as shown in Figure 4, the connection between the face sheet and core exhibits excellent continuity, thus avoiding secondary connection typically needed during conventional processing. Note that all the test specimens listed in Table 1 were fabricated using the method of additive manufacturing. Due to limitations of the additive manufacturing facility used in the current study, relatively large size errors were found in specimens having either thin face sheets (e.g., Specimen VII) or thin core web sheets (e.g., Specimen I). For instance, from the SEM image displayed in Figure 4, partially non-molten particles (0.02–0.03 mm in size) are present on the surface of core web sheet, causing large roughness of the surface. This is the main reason behind the large relative size offsets between the as-fabricated specimens and the envisioned specimens shown in Table 3.

**Figure 5:**
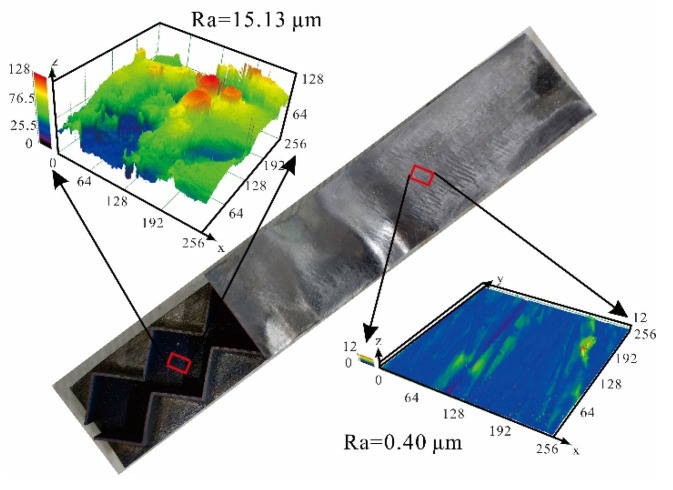
Typical surface characteristics of as-fabricated Ti-6Al-4V alloy 3CSP specimen.

Contrasted with specimens fabricated using the traditional methods such as by vacuum brazing, surface property has become a focal point in the evaluation of processing quality for specimens fabricated with additive manufacturing [24,26]. Therefore, surface roughness on the face sheets of as-fabricated specimens with/without polishing treatment was characterized using a confocal microscope (OLYMPUS 3D measuring laser microscope OLS4000; Olympus Corporation, Shinjuku, Japan). As illustrated in Figure 5, the roughness (R_a_) of the outer surface that was polished is 0.40 µm, while that of the in-situ inner surface without polishing is 15.13 µm. Further analysis of the unpolished surfaces revealed non-molten powders deposited on these surfaces, thus causing roughened surfaces of face sheets and core web plates, as shown by the SEM image of Figure 4b. The surface roughness results in the relative size offsets between the as-fabricated and envisioned specimens shown in Table 3. These size offsets are one of the reasons for the relatively large deviations of the present analytical/numerical predictions from experimental measurements. Previous studies showed that reasonable surface treatment such as surface polishing contributes to the removal of non-molten parent powders [24] and the reduction of surface roughness [27], leading to improved constitutive mechanical behavior at microscopic level. Likewise, the influence of non-molten residuum on the bending performance of a 3D printed specimen is obvious [28].

#### 2.1.3. Material Properties of Parent Material

To characterize the mechanical properties of the parent material, dog-bone shaped tensile specimens of Ti-6Al-4V were manufactured with SLM 3D printing. To ensure identical mechanical properties, the dog-bone tensile specimens and the 3CSP test specimens for 4-point bending were fabricated simultaneously. Tensile tests were performed using a standard servo-hydraulic test machine (MTS-858 Mini bionix; MTS Corporation, Eden Prairie, MN, USA) at ambient temperature, with a nominal strain rate set as 1 × 10^−3^ s^−1^ in reference to ISO 6892-1:2009 [29]. The load and the strain were measured by the loading gauge with an accuracy of 1 N and an extensometer with an accuracy of 10^−3^ mm, respectively.

**Figure 6:**
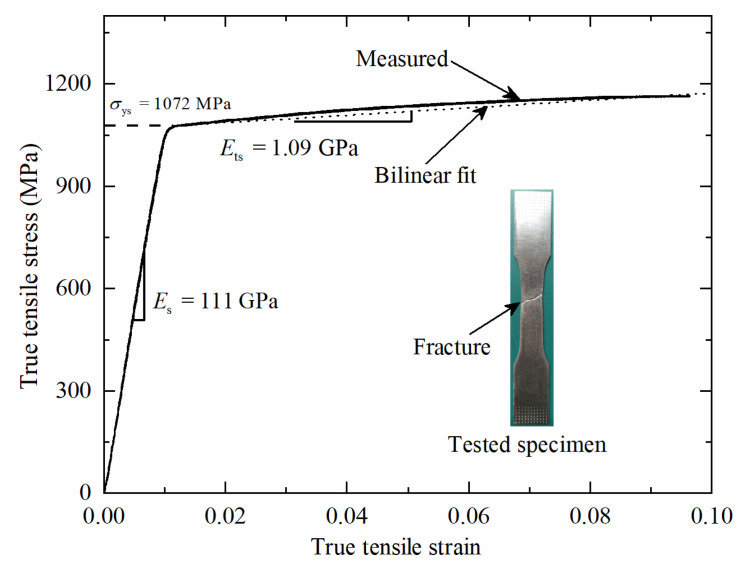
Uniaxial true stress versus true strain curve of 3D-printed Ti-6Al-4V alloy.

Quasi-static uniaxial true strain versus true stress curve measured using the dog-bone shaped Ti-6Al-4V specimen is presented in Figure 6. It is shown that the parent material obtained with SLM 3D printing can be expressed approximately as an elastic-plastic material, with a Young’s modulus of *E*_s_ =111 GPa, a yield strength of *σ*_ys_ =1072 MPa, and a linearly hardening modulus of *E*_ts_ =1.09 GPa. These measured material properties of Ti-6Al-4V were subsequently applied to analytical predictions and finite element (FE) simulations, as detailed in the following sections.

### 2.2. Experimental Setup for Four-Point Bending

**Figure 7:**
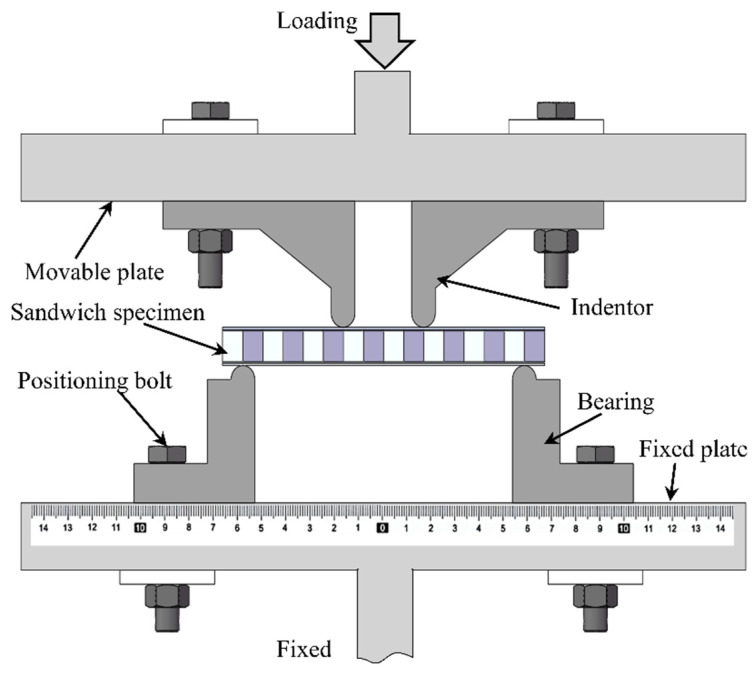
Set-up for four-point bending test.

Both four-point bending and three-point bending tests are widely used experimental methods for assessing the bending response of a structure. Compared with three-point bending test, four-point bending test produces a pure bending region between the two indenters, thus reducing stress concentration in the region in close contact with the indenter. In the current study, the four-point bending experiment setup is illustrated in Figure 7. The sandwich specimen was placed between the indenters and bearings, both having a diameter of 10 mm. Four-point bending test was performed with an MTS-880 experimental loading system (MTS Corporation, Eden Prairie, MN, USA). With reference to testing codes ASTM C393-11 and D7249, the loading rate was fixed at 0.5 mm/min to mimic quasi-static loading. Analog signals for the load and displacement data were extracted by the measure sensors of the MTS system. To capture the deformation and collapse evolution of each specimen, a high-resolution photography system was utilized to record the entire experiment process.

### 2.3. Finite Element Simulation

Finite element (FE) simulations were performed with ABAQUS v6.10 (Dassault Systèmes, Vélizy-Villacoublay, France). Geometries of the FE model were identical to those of the test specimen. Ti-6Al-4V was modeled as an isotropic hardening material, as characterized in Section 2.3, with a Young’s modulus of *E*_s_ = 111 GPa, a Poisson ratio of ν_s_ = 0.34, and a yield stress of *σ*_ys_ = 1072 MPa (Figure 6). Both the face sheets and core webs of the sandwich specimen were meshed using four-node shell elements (S4R), with their dimensions set not according to the designated values but to the measured sizes listed in Table 3. As a result, relative size offsets and deviations were accounted for in the FE simulations. Each 3CSP specimen was made and integrated with 3D printing, requiring no secondary connection as in the construction of sandwich structures using traditional methods. From the SEM image shown in Figure 4a, it can be seen that, with additive manufacturing, no interaction layer is present between the core web and the face sheet. Therefore, in the present FE simulations, the face sheet and the core web were merged into one component using the Boolean operation: that is, it was assumed that the core web is perfectly bonded to the face sheet. It should be mentioned that, at present, there is not yet a report on the Poisson ratio of 3D printed titanium alloy. In the current study, as an approximation, we simply used the Poisson ratio (0.34) measured for traditional rolled titanium alloy sheets [20]. In a future study, we plan to evaluate systematically how 3D printing and the associated processing parameters affect the Poisson ratio of titanium alloys.

**Figure 8:**
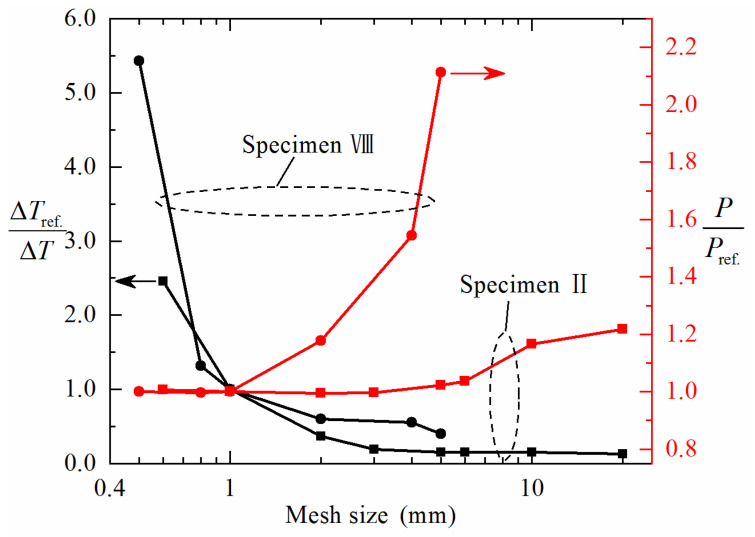
Mesh insensitivity on initial failure load and computational efficiency for Specimens II and VIII.

The indenters and bearings were modeled as 3D analytical rigid shells, as they are much stiffer than the sandwich specimen. A fixed displacement boundary condition was used for each bearing. An automatic face-to-face interaction algorithm based on the penalty function was used to simulate the contacts between indenter/bearings and face sheets, with the friction coefficient in the tangential direction fixed at 0.1. The selection of a meshing size of ~1 mm was determined after performing a systematic study of mesh convergence, as further refinement is found to have negligible effect on numerically simulated four-point bending responses. For Specimens II and VIII, Figure 8 plots the numerically calculated initial failure load *P*, normalized by $P_{\mathrm{ref}.}$ calculated with the mesh size fixed at 1 mm, as a function of mesh size. Similar results were obtained for other specimens and hence not presented here for brevity. It is shown that the value of $P/P_{\mathrm{ref}.}$ converges when the mesh size drops to 3 mm for Specimen II and 1 mm for Specimen VIII. Meanwhile, the stable time increment ΔT is also plotted in Figure 8, where $\Delta T_{\mathrm{ref}.}$ is the stable time increment estimated with 1 mm mesh size. The larger is the $\Delta T_{\mathrm{ref}.}/\Delta T$, the lower is the computational efficiency. Thus, with the numerical convergency and computational efficiency considered simultaneously, the optimal mesh size in this study was selected as 1 mm.

To simulate quasi-static bending with initial imperfections, the present FE simulations were conducted with linear perturbation and explicit dynamics solvers. A buckling eigenvalue analysis was first performed to select the initial geometry imperfection pattern for subsequent quasi-static four-point bending simulation. Upon performing a velocity independence analysis, the velocity of the indenters was set to be 0.1 m/s.

### 2.4. Analytical Modeling

This section describes how the flexural stiffness and initial collapse load of the present 3CSP sandwich structure can be predicted using analytical models. Following the classical work of Allen [30], it was assumed that the face sheets are mainly subjected to bending moments, while the cores are mainly subjected to transverse shear. To make the prediction theoretically valid, none of the specimens in this study was allowed to exceed the geometric threshold for normalized core height [18].

#### 2.4.1. Bending Stiffness

The total deflection δ of a sandwich structure under four-point bending can be expressed as a linear superposition of deflection *δ*_M_ caused by the bending moment and deflection *δ*_V_ caused by the shear force, namely:

(1) $$\delta =\delta_{M}+\delta_{V}$$

The deflection caused by the bending moment is calculated as [31]:

(2) $$\frac{\delta_{M}}{P}=\frac{{\left(L_{b}-\chi \right)}^{2}\chi^{2}}{6L_{b}D_{\mathrm{eq}}}+\frac{\chi }{12L_{b}D_{\mathrm{eq}}}\left[\frac{L_{b}}{\chi }{\left(2\chi -L_{b}\right)}^{3}+\left(L_{b}{}^{2}-2\chi^{2}\right)\chi \right]$$

where *P* is the total force, half of which is transferred to the loaded specimen by each indenter. Let $D_{\mathrm{eq}}$ denote the equivalent flexural rigidity of the sandwich.

In practice, the corrugated channel core is in general not loaded in the transverse direction (*y*-direction in Figure 1) [32]. Therefore, contribution of the core to the flexural rigidity may be ignored. The equivalent flexural rigidity $D_{\mathrm{eq}}$ can thence be expressed as:

(3) $$D_{\mathrm{eq}}=E_{s}\frac{Bt_{f}{}^{3}}{6}+E_{s}\frac{Bt_{f}h^{2}}{2}$$

where *E*_s_ is the Young’s modulus of the face sheet material.

In a simply supported beam subjected to a total force *P*, the shear force in the region between the left indenter and the left bearing is *P*/2. Thus, the deflection caused by the shear force is determined as:

(4) $$\frac{\delta_{V}}{P}=\frac{\chi }{2G_{\mathrm{eq}}Bh}$$

where represents the equivalent shear modulus of the core. Its value can be calculated as:

(5) $$G_{\mathrm{eq}}=\bar{\rho }G\cos^{2}\theta$$

where $\bar{\rho }=\frac{t_{c}}{d\cos\theta }$ is the relative density of the corrugated channel core and *G* denotes the shear modulus of the parent material of the core.

#### 2.4.2. Initial Failure Loads

Under four-point bending, a 3CSP specimen may exhibit four major collapse modes: (i) face yielding (FY); (ii) face buckling (FB); (iii) core yielding (CY); and (iv) core buckling (CB). The pure bending section of the specimen is subjected to the largest bending moment, given by:

(6) $$M=\frac{P\chi }{2B}$$

where *M* represents the bending moment per width. As mentioned above, it was assumed that the bending moment is carried by the face sheets. Therefore, the maximum normal stress on the loaded face sheets is:

(7) $$\sigma_{f}=\frac{P\chi }{2Bt_{f}\left(h+t_{f}\right)}$$

At the same time, the maximum shear force per width in the loaded core is determined as:

(8) $$V=\frac{P}{2B}$$

such that the maximum shear stress in the loaded core is:

(9) $$\tau_{c}=\frac{Pd}{2Bt_{c}h}$$

Correspondingly, the initial failure criteria of each collapse mode are summarized as shown below. The critical stress of face yielding is:

(10) $$\sigma_{f}=\sigma_{\mathrm{ys}}$$

where $\sigma_{\mathrm{ys}}$ is the tensile yield strength of the parent material. It follows that the face yielding load is:

(11) $$P_{\mathrm{FY}}=\frac{2Bt_{f}\left(h+t_{f}\right)}{\chi }\sigma_{\mathrm{ys}}$$

At the onset of core yielding, the critical load is:

(12) $$\tau_{c}=\tau_{\mathrm{ys}}$$

where $\tau_{\mathrm{ys}}$ is the shear yielding strength of the parent material, which was assumed to depend upon the tensile yield strength as $\tau_{\mathrm{ys}}=\sigma_{\mathrm{ys}}/\sqrt{3}$. Correspondingly, the core yielding load is determined as:

(13) $$P_{\mathrm{CY}}=\frac{2Bt_{c}h}{d}\tau_{\mathrm{ys}}$$

The critical stress of face buckling is given by:

(14) $$\sigma_{\mathrm{fb}}=\frac{k_{\mathrm{fb}}\pi^{2}E_{s}}{12\left(1-\nu_{s}{}^{2}\right)}{\left(\frac{t_{f}}{d}\right)}^{2}$$

where *E*_s_ and $\nu_{s}$ denote the Young’s modulus and Poisson ratio of the parent material, respectively. *k*_fb_ is the compression buckling coefficient, approximately equal to 6.97. Face buckling will not take place if $\sigma_{f}\leq \sigma_{\mathrm{fb}}$. Hence, the critical load of face buckling is given by:

(15) $$P_{\mathrm{FB}}=\frac{2Bt_{f}\left(h+t_{f}\right)}{\chi }\sigma_{\mathrm{fb}}$$

The shear buckling stress induced by core buckling is:

(16) $$\tau_{\mathrm{cb}}=\frac{k_{\mathrm{cb}}\pi^{2}E_{s}}{12\left(1-\nu_{s}{}^{2}\right)}{\left(\frac{t_{s}}{h}\right)}^{2}$$

where *k*_cb_ is the shear buckling coefficient, which depends on the geometry parameter *h*/*s*. For a rectangular core panel with an aspect ratio of *h*/*s* =1, the value of *k*_cb_ is assumed to be 14.71. Core buckling occurs when $\tau_{c}=\tau_{\mathrm{cb}}$. Therefore, the critical load of core buckling is calculated as:

(17) $$P_{\mathrm{CB}}=\frac{2Bt_{c}h}{d}\tau_{\mathrm{cb}}$$

## 3. Results and discussion

In this section, quasi-static four-point bending results obtained from analytical predictions, FE simulations, and experimental measurements are summarized and compared. Let the non-dimensional bending stiffness be represented by $S=\bar{P}/\bar{\delta }$, where $\bar{P}=P/\left(2E_{s}B\chi \right)$ is the non-dimensional initial failure load and $\bar{\delta }=\delta /\chi$ is the corresponding non-dimensional indenter displacement.

### 3.1. Observations of Structural Failure

#### 3.1.1. Core Yielding

**Figure 9:**
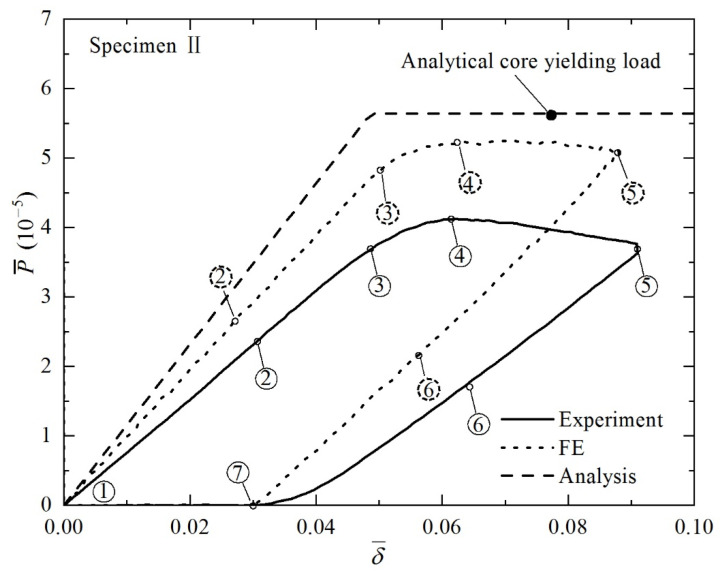
Non-dimensional load versus non-dimensional indenter displacement curve of Specimen II under four-point bending. Numbers circled by solid and dashed lines represent typical phases of structural responses observed in experiment and FE simulation, respectively. Corresponding deformed specimen configurations are displayed in Figure 10.

**Figure 10:**
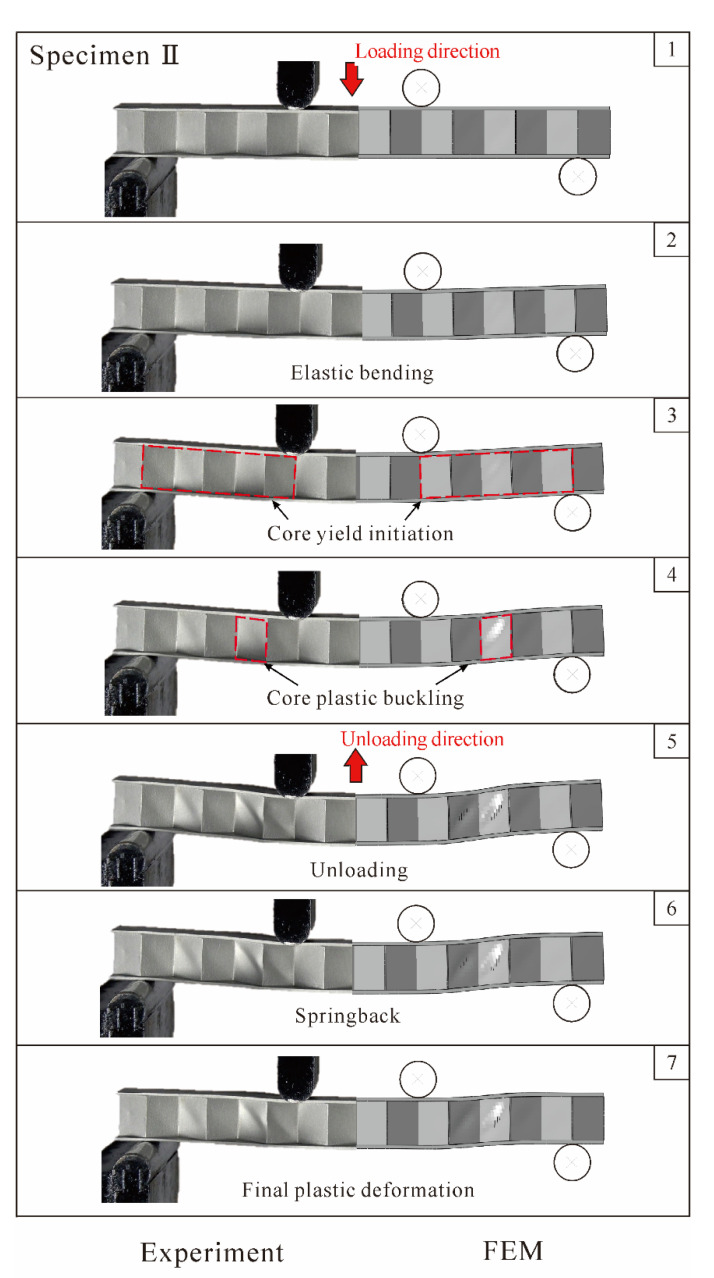
Comparison between numerically simulated deformation and failure patterns of Specimen II with those observed during experiment. Circled numbers correspond to those marked on load versus deflection curves of Figure 9.

Figure 9 displays the experimentally measured, analytically predicted, and numerically simulated load versus deflection curves for 3CSP Specimen II (Table 1): note that the initial failure of this specimen is core yielding, as indicated by the analytical predictions. Corresponding to the circled numbers on the curves, photos of the deformed specimen are compared in Figure 10 with those numerically simulated. It should be pointed out that, during the present experiments, the method of synchronous triggering is used to ensure that data collection by the testing machine and image acquisition by the photography system have identical zero time. Thus, for any point on the load versus displacement curve, the exact photo image corresponding to this point can be extracted, thus enabling the comparisons shown in Figure 10 and Figure 12. The loading procedure is marked as Points 1–5, while the unloading as Points 6 and 7. As Figure 9 illustrates, during loading, the bending response of Specimen II is composed of three major phases: (i) the elastically loading phase (1–3); (ii) the plastically stable phase (3–4); and (iii) the plastic buckling phase (4–5). The results in Figure 9 and Figure 10 show that the key features obtained with FE simulations agree with corresponding experimental observations. In the elastically loading phase, the initial failure load $\bar{P}$ increases linearly with indenter displacement $\bar{\delta }$. The specimen exhibits global bending, with no obvious out of plane deformation in the face sheets and core webs, as shown in Sequence 2 of Figure 10. With further loading, a plastic stable phase emerges, represented by 3 and 4 in Figure 9, wherein a gradual reduction in stiffness occurs, and the load reaches a peak at Point 4. From the corresponding Image 3 shown in Figure 10, the premature collapse initiation of core yielding can be seen in the core shear region between the indenter and the bearing. As the loading continues, shear deformation in the core becomes more visible in Image 4 of Figure 10. Then, the plastic buckling response begins and the load decreases with increasing indenter displacement. Subsequently, an unloading procedure is performed. The corresponding response is shown on the experimental and simulation curves as 5–7. During unloading, as elastic strain energy is released, springback of the specimen occurs, the radius of curvature becomes larger, and the deformation decreases. When the load $\bar{P}$ is dropped to 0 N, permanent plastic deformation characterized with core yielding dominates the specimen.

#### 3.1.2. Face Yielding

**Figure 11:**
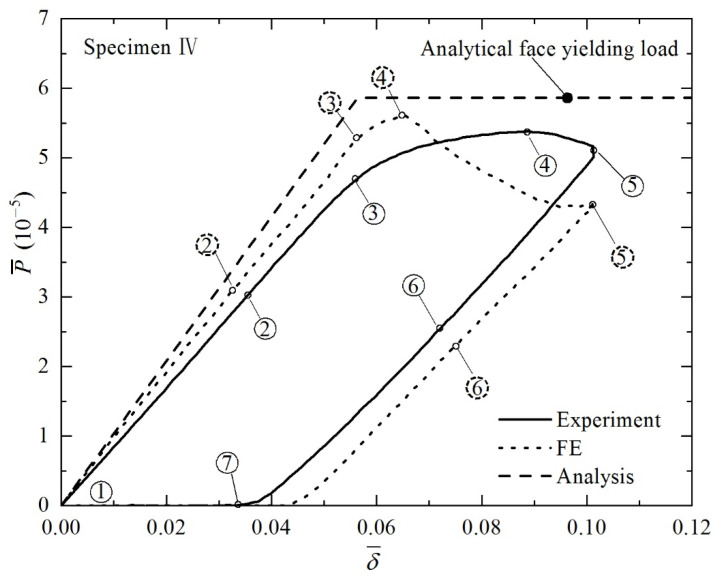
Non-dimensional load versus non-dimensional indenter displacement curve of Specimen IV under four-point bending. Numbers circled by solid and dashed lines represent typical phases of structural responses observed in experiment and FE simulation, respectively. Corresponding deformed specimen configurations are displayed in Figure 12.

**Figure 12:**
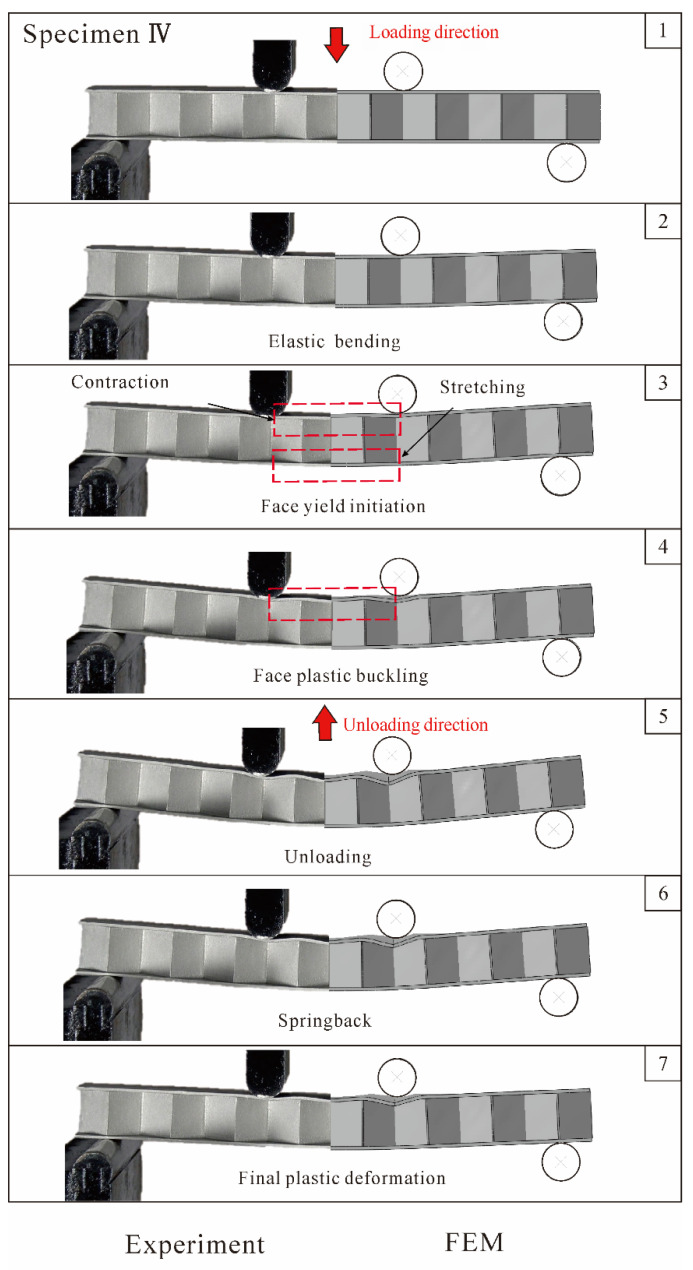
Comparison between numerically simulated deformation and failure patterns of Specimen IV with those observed during experiment. Circled numbers correspond to those marked on load versus deflection curves of Figure 11.

Similar to the case of core yielding, load versus displacement curves and corresponding deformed configurations are presented in Figure 11 and Figure 12 for the case of face yielding (Specimen IV in Table 1). Again, the bending response can be characterized into three phases. After a linearly progressive elastic phase, plastic deformation dominates with the initiation of face yielding, as indicated on Images 3 and 4 of Figure 12. The plastic deformation is accompanied by stretching of the bottom face sheet and contraction of the top face sheet in the pure bending region. Unlike Specimen II, the core of Specimen IV experiences minimal plastic deformation, while its face sheets undergo dramatically plastic deformation. The top face sheet buckles, developing a number of waves, as shown in Image 5 of Figure 12. For Specimens VI, the FE simulation results agree well with those of the experiments, but the analytical predictions somewhat overestimate due to idealized assumptions made in the modeling.

#### 3.1.3. Face Buckling

**Figure 13:**
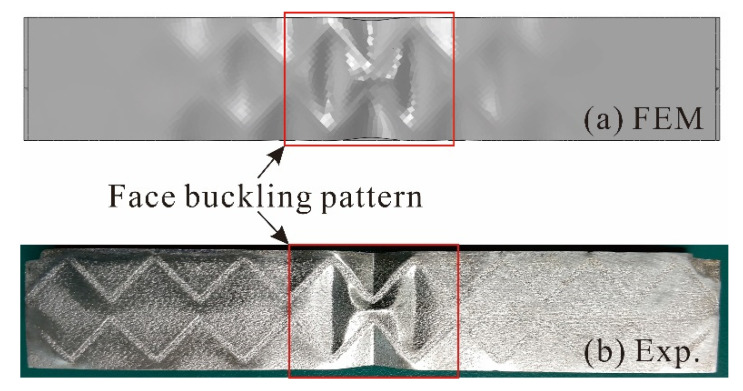
Final deformation pattern of Specimen VII: comparison between (**a**) FE simulation and (**b**) experiment.

**Figure 14:**
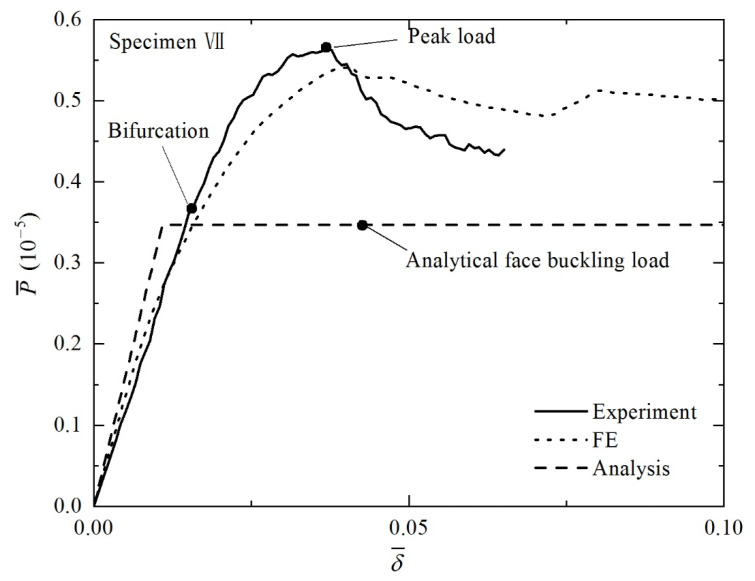
Non-dimensional load versus non-dimensional indenter displacement curves of Specimen VII.

In addition to the collapse modes discussed above for Specimens II and IV, face buckling is captured in Specimen VII, both experimentally and numerically. Its final deformation patterns and load versus deformation curves obtained via experiment and FE simulation are displayed in Figure 13 and Figure 14. Good agreement is obtained between numerical and experimental results for bending stiffness and initial failure load. The elastic buckling of a plate is well known to exhibit a stable post buckling response [33]. Therefore, the peak loads obtained from the present experiment and simulation are much larger than that predicted analytically, as shown in Figure 14.

### 3.2. Comparison among Experimental, Analytical and FE Results

For each 3CSP specimen listed in Table 1, Table 4 compares the experimental, analytical, and numerical (FE) results obtained for its non-dimensional bending stiffness, non-dimensional initial failure load, and collapse modes. It is shown that, when contrasted with experimental measurements, both the analytical and FE models predict accurately the failure modes but overestimate the bending stiffness and initial failure load. For the analytical model, the prediction error ranges 18–41%, whereas for the FE model, the error ranges 5–26%. Further, it is found that the load-carrying capacity of a 3CSP structure depends strongly on its structural geometry. To explore the underlying mechanisms, three typical experiments with different failure modes are analyzed in detail next.

### 3.3. Discussion

#### 3.3.1. Effect of Geometric Sizes on Bending Stiffness

The results in Table 4 reveal that relatively large discrepancies exist between experimentally measured and numerically calculated bending stiffnesses and initial failure loads: for instance, for Specimen III, the discrepancy of bending stiffness is 26%. This is mainly attributed to geometric imperfections induced during 3D printing. According to the specific morphology of the corrugated channel core, the method of SLM involves three main steps.

1. Printing: To ensure the face sheet and the core are formed in one step, the specimen needs to be placed at an inclined angle, with a support used between the supporting plate and the suspended surface to ensure the forming accuracy.
2. Heat treatment: To eliminate residual stresses induced during 3D printing, the as-printed specimen (together with the supporting plate) is put into a vacuum furnace, with the furnace temperature controlled in accordance with that shown in Figure 3.
3. Post processing: Upon removing the supporting plate via wire cutting, electrical grinding tool and sandpaper are used to manually polish the surfaces of the specimen.

**Figure 15:**
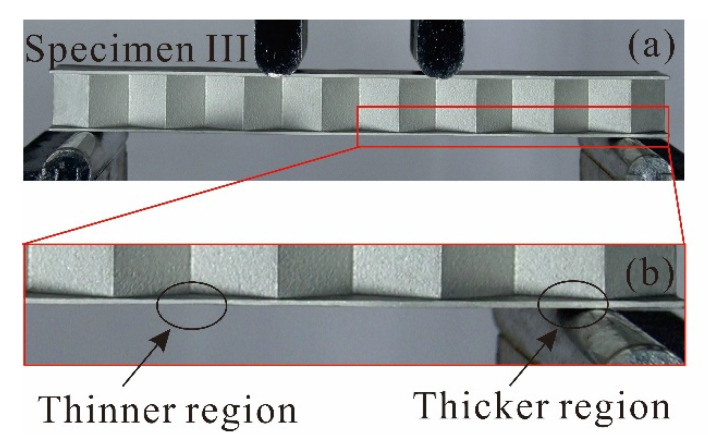
Geometric imperfections of Specimen III: (**a**) lateral view of Specimen III before experiment; and (**b**) the partial enlarged view of Specimen III.

Taking again Specimen III as an example, because the processing accuracy of the present wire cutting and surface polishing methods is not high, its face sheets are not even, as shown clearly in Figure 15. In the present study, the analytical model assumes that both the top and bottom face sheets of a 3CSP specimen have uniform thickness. Therefore, in accordance with the analytical model, the face sheet thickness listed in Table 3 for each specimen is the average of measurements at selected positions along the face sheet. This causes the discrepancy between model prediction and experimental measurement. Further, in the FE simulations, shell elements having uniform thickness are used to model the face sheet, which also leads to discrepancy between numerical simulation and experimental measurement. In future studies, 3D solid elements in lieu of shell elements will be employed to refine the FE model so as to quantify the influence of non-even face sheets on the mechanical behavior of a 3CSP structure.

**Figure 16:**
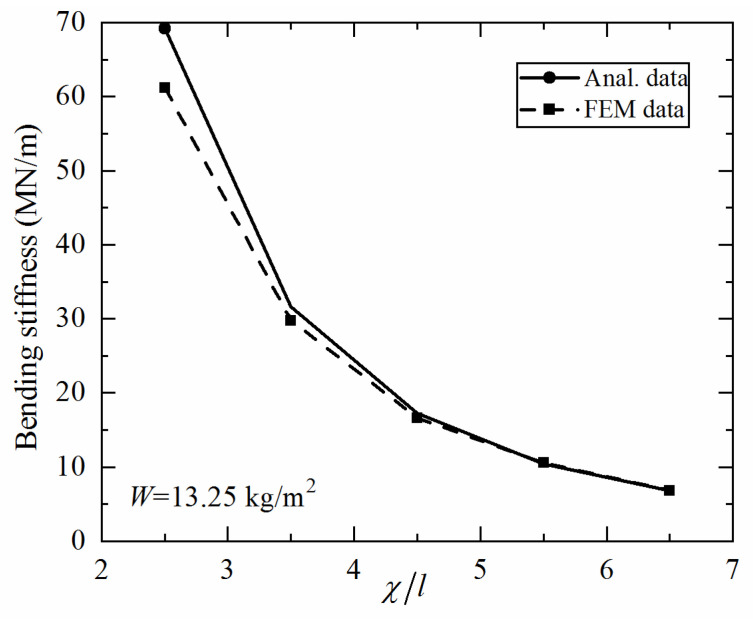
Effect of wave numbers of 3CSP sandwich panels with *W* = 13.25 kg/m^2^ on bending stiffness.

When developing the present analytical model, the bending deformation of a 3CSP specimen is taken as the superposition of deformation caused by the bending moment and that induced by the shear force. The former is dictated by the bending stiffness of the face sheets, while the latter is dominated by the shear stiffness of the core. The equivalent shear stiffness of the core, as shown in Equation (5), is derived via homogenization, which requires that a sufficiently large number of cells are present within the characteristic length of the 3CSP structure [34]. In the current study, due to 3D printing accuracy and constraints on printed cell size, the ratio of characteristic length to unit cell length is χ/l=2.5 for each specimen. This causes the discrepancy between analytical model and FE simulation. Figure 16 plots the analytically predicted bending stiffness as a function of χ/l for specimens having identical total mass ($W=13.25\;\mathrm{kg}/m^{2}$). For comparison, results calculated with the FE model are also presented. It is shown that the two curves converge only when the value of χ/l is sufficiently large, e.g., at χ/l=5.5. Therefore, to ensure the prediction accuracy of analytical modeling for 3CSP structures, special focus needs to be placed upon the magnitude of χ/l in future studies.

#### 3.3.2. Effect of Poisson Ratio

**Figure 17:**
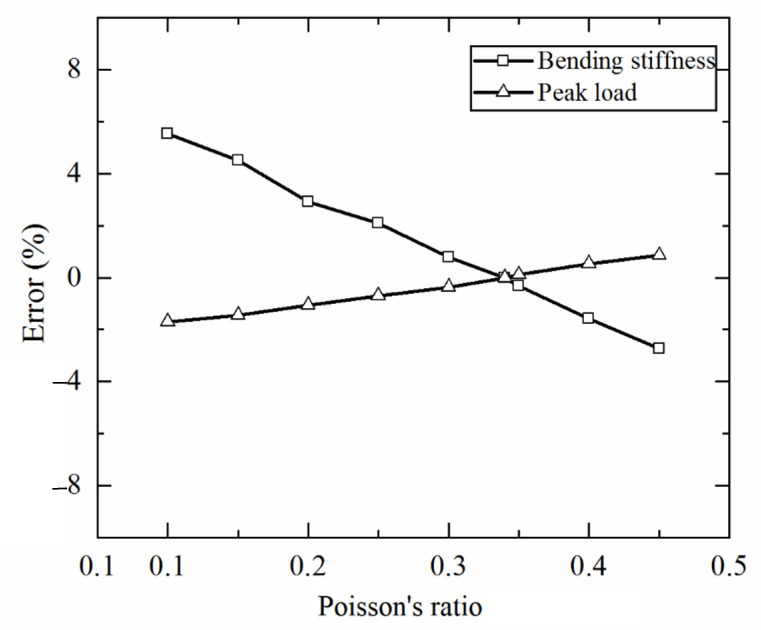
Effect of Poisson ratio on numerically calculated bending stiffness and peak load of 3CSP structure.

As mentioned above, in the absence of Poisson ratio for 3D printed Ti-6Al-4V, a Poisson ratio of 0.34 measured using traditional rolled titanium alloy sheets is used in the present study. In this section, how the bending response of a 3CSP structure is dependent upon the value of Poisson ratio is quantified using the FE model. As the value of Poisson ratio is varied in the range of 0.10–0.45, Figure 17 presents the errors of numerically calculated bending stiffness and peak load relative to the reference case of Poisson ratio equaling to 0.34. It is shown that, for the bending stiffness, the largest error of 5.54% occurs when the Poisson ratio is 0.1. Similarly, for the peak load, the largest error of 1.7% also occurs when the Poisson ratio is set to 0.1. Overall, the influence of Poisson ratio on the numerically simulated mechanical performance of 3CSP structures is small.

## 4. Collapse Mechanism Maps

Collapse mechanism maps [35,36] are used next to probe the effect of structural geometry on collapse initiation mode. To this end, dimensionless quantifications of structural response are conducted for the cases described in the previous section, with normalized geometrical parameters introduced as:

(18) $${\bar{t}}_{f}=\frac{t_{f}}{\chi },\;{\bar{t}}_{c}=\frac{t_{c}}{\chi },\;\bar{h}=\frac{h}{\chi },n=\frac{d}{h}$$

Upon substituting these normalized parameters into Equations (11), (13), (15), and (17), the critical loads corresponding to the four collapse modes can be rewritten as:

(19) $${\bar{P}}_{\mathrm{FY}}=\varepsilon_{y}{\bar{t}}_{f}(\bar{h}+{\bar{t}}_{f})$$

(20) $${\bar{P}}_{\mathrm{FB}}=\frac{k_{f\;b}\pi^{2}}{12(1-\nu_{s}{}^{2})}{\left(\frac{1}{n\bar{h}}\right)}^{2}{\bar{t}}_{f}{}^{3}(\bar{h}+{\bar{t}}_{f}),$$

(21) $${\bar{P}}_{\mathrm{CY}}=\varepsilon_{y}\frac{{\bar{t}}_{c}}{n\sqrt{3}},$$

(22) $${\bar{P}}_{\mathrm{CB}}=\frac{k_{\mathrm{cb}}\pi^{2}}{12(1-\nu_{s}{}^{2})}\frac{{\bar{t}}_{c}{}^{3}}{n{\bar{h}}^{2}},$$

where $\varepsilon_{y}=\sigma_{y}/E_{s}$ is the yielding strain.

**Figure 18:**
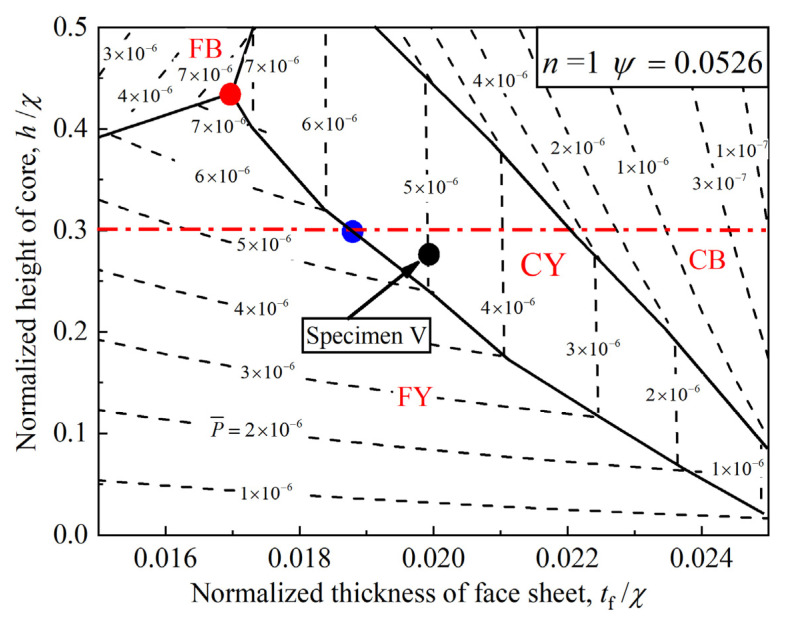
Prototypical collapse map for 3CPS structure and the corresponding optimal design strategy for the scenario of n=1 and ψ=0.0526. Geometric threshold of core height is represented by dash-dotted line.

To facilitate visualization of optimization results based on failure maps, Figure 18 plots a prototypical collapse map for the scenario of n=1 and ψ=0.0526, where “FB” “FY”, “CB”, and “CY” represent abbreviations of the four collapse modes. “F” and “C” represent “Face sheet” and “Core”, while “Y” and “B” represent “Yielding” and “Buckling”, respectively. Non-dimensional initial failure load contour plots for the 3CSP structure with different geometries are delineated via the dashed lines. With these contour plots, the optimal geometries can be obtained by searching for those associated with higher $\bar{P}$ values. Optimal geometries thus obtained are marked with and without considering geometry limitation, represented by red and blue solid circles, respectively. For a 3CSP specimen with ψ=0.0526, excellent bending resistance can be achieved by a geometry lying at the intersection point (red solid circle) of face buckling, face yielding, and core yielding, if geometrical limitation is ignored. The optimal geometry lies on the confluence (blue solid circle) of face yielding, core yielding, and geometric limitation (if geometric limit is considered). Further, given the geometric parameters of Specimen V in Table 1, its bending resistance can be effectively enhanced by decreasing the normalized thickness of face sheet and increasing the normalized height of core (black solid circle in Figure 18).

**Figure 19:**
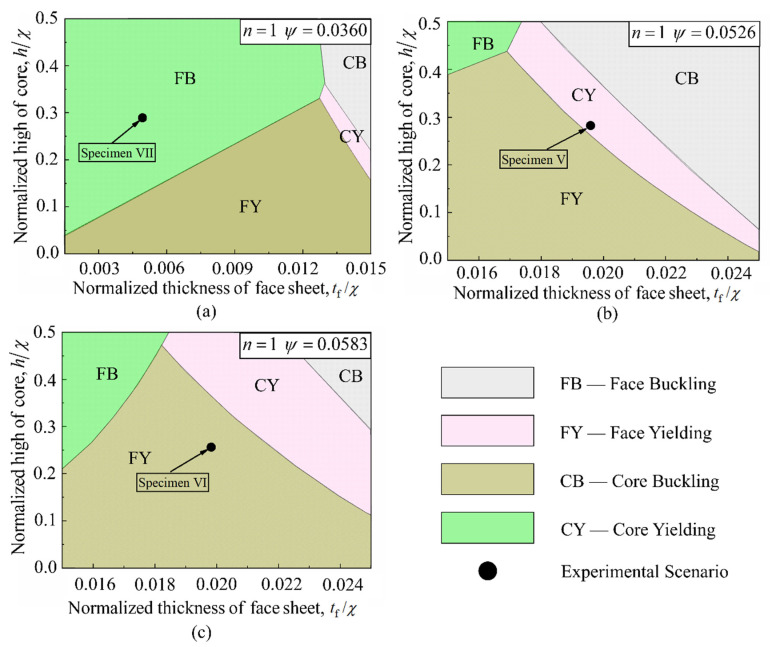
Prototypical collapse maps for each 3CSP structure with: (**a**) ψ=0.0363; (**b**) ψ=0.0526; and (**c**) ψ=0.0583. In all cases, the alternative key parameter was fixed at *n* = 1.

Figure 19 presents the collapse maps for three 3CSPs having different values of ψ. For the given range of normalized geometries, the initial collapse modes for sandwich structures with different geometries (characterized with different values of n and ψ) vary. Solid circles marked in Figure 19 represent the geometries of experimental specimens described separately in the previous section, namely Specimens V, VI, and VII. Regardless of the geometry of a 3CSP specimen, the four competing collapse modes can all occur. For the scenario of n=1 and ψ=0.0363, the dominating initial collapse modes are collapses produced from the face sheets, which can be validated via the four-point bending test of Specimen VII. Within the ${\bar{t}}_{f}$ range of 0.001–0.015 and $\bar{h}$ range of 0.0–0.3, the collapse mechanism mode is presented by face sheet bucking and yielding when ${\bar{t}}_{f}$ is less than 0.013. However, when ${\bar{t}}_{f}$ exceeds 0.013, the collapse mechanism mode changes from face sheet yielding to core yielding and core buckling. Between these two modes of core collapse, core buckling increasingly dominates as the core height is increased.

The domination of collapse mode(s) also varies with varying structural mass. As the value of ψ is increased, the region representing core collapse grows, as shown in Figure 19a–c. For the scenario of n=1 and ψ=0.0526 (Figure 19b), the component of the collapse mechanism map assembles with the four competing collapse modes: i.e., face sheet buckling, face sheet yielding, core buckling, and core yielding, consistent with the scenario of n=1 and ψ=0.0583 (Figure 19c). However, Figure 19b,c shows that the core buckling and core yielding collapse regions gradually expand relative to those of Figure 19a. Additionally, the solid circles marked in each collapse mechanism map of Figure 19 reveal that the 3CSP specimens tested in this study do not have optimal geometries.

It must be pointed out that the present analytical predictions deviate, in some cases quite significantly, from experimental results. As previously discussed, these large discrepancies are likely caused by the idealized assumptions (e.g., sufficiently large number of unit cells to ensure the prediction accuracy of homogenization) made to simplify the analytical modeling, as well as by the relatively low surface quality of 3CSP specimens fabricated using the 3D printing facility at hand. As a preliminary study, while the idealized analytical model was employed to carry out the current structural optimization, the results need be applied in caution, especially when the core height is large where the intersection points may shift by as much as 20–30%. More comprehensive and accurate collapse mechanism maps will be constructed in future studies with significantly improved analytical models.

## 5. Conclusions

Novel ultra-lightweight corrugated-channel-cored sandwich structures are envisioned and fabricated from Ti-6Al-4V alloy using the selective laser melting (SLM) methodology. Their performances (e.g., bending resistance and initial collapse modes) when subjected to four-point bending are subsequently experimentally measured. A combined approach of analytical modeling and numerical simulation based on the method of finite elements (FE) is employed to further explore in detail physical mechanisms underlying the bending performance. The main conclusions are summarized as follows.

1. Four competing collapse initiation modes, i.e., face-sheet yielding, face-sheet buckling, core yielding, and core buckling, govern the failure processes of a 3CSP structure as its geometry is varied.
2. Both the analytical and FE models predict accurately the failure modes but overestimate the bending stiffness and initial failure load.
3. Collapse mechanism maps constructed using the developed analytical model provide an effective strategy for designing 3CSP structures with optimal bending responses.
4. The collapse mechanism maps can be employed to quantify the influence of 3CSP structural topology on collapse initiation modes.

The proposed 3CSP structures hold great potential in a wide variety of multifunctional applications targeting simultaneous load-bearing, and heat dissipation via active cooling at ultra-lightweight. Nonetheless, several issues of the present work need to be addressed in future studies, including the relatively large deviations of analytical and FE predictions from experimental measurements, the determination of Poisson ratio for 3D printed titanium alloys, the improvement in processing quality with 3D printing, and the characterization of heat transfer performance of 3CSP structures.

## Figures and Tables

**Table 1:** Geometric details of sandwich panels with corrugated channel cores.

Specimen No.	Length *L* (mm)	Width *W* (mm)	Core Height *h* (mm)	Web Spacing *d* (mm)	Thickness of Face Sheet *t*_f_ (mm)	Thickness of Core Sheet *t*_c_ (mm)	Angle *θ* (°)	Wave Length *l* (mm)
I	136	24	12	12	1.0	0.4	45	17
II	136	24	12	12	1.0	0.4	45	17
III	136	24	12	12	1.0	0.6	45	17
IV	136	24	12	12	0.8	0.6	45	17
V	136	24	12	12	0.8	0.4	45	17
VI	136	24	12	12	0.8	0.6	45	17
VII	136	24	12	12	0.2	0.8	45	17

**Table 2:** SLM processing parameters for Ti-6Al-4V alloy 3CSP specimens.

Term	Layer Thickness	Laser Powder	Scan Speed	Hatch Distance	Particle Size
Parameter	40 μm	230 W	970 mm/s	100 μm	15–53 μm

**Table 3:** Measured dimensions of test specimens fabricated with the SLM.

Specimen No.	Thickness of Face Sheet (mm)			Thickness of Core Sheet (mm)		
	Design Size	Measured Size	Error	Design Size	Measured Size	Error
I	1.0	0.992	−0.80%	0.4	0.445	11.25%
II	1.0	1.063	6.30%	0.4	0.430	7.50%
III	1.0	1.042	4.20%	0.6	0.645	7.50%
IV	0.8	0.853	6.62%	0.6	0.551	−8.17%
V	0.8	0.831	3.87%	0.4	0.407	1.75%
VI	0.8	0.813	1.62%	0.6	0.603	0.50%
VII	0.2	0.225	12.50%	0.8	0.764	−4.50%

**Table 4:** Summary of four-point bending respons es from analytical prediction, FE simulation, and experimental measurement.

Specimen No.	Bending Stiffness *S* (10^−5^)			Initial Failure Load $\bar{P}$ (10^−5^)			Collapse Mode		
	Analysis	Simulation	Experiment	Analysis	Simulation	Experiment	Analysis	Simulation	Experiment
I	111.11	99.06	76.91	5.84	5.34	3.72	CY	CY	CY
II	116.00	97.41	77.57	5.64	4.87	3.76	CY	CY	CY
III	129.93	103.12	76.42	7.27	5.46	3.58	FY	FY	FY
IV	104.51	94.55	85.79	5.86	5.26	4.54	FY	FY	FY
V	93.51	80.82	76.19	5.34	4.29	3.74	CY	CY	CY
VI	102.53	90.55	82.82	5.57	5.11	4.65	FY	FY	FY
VII	31.79	22.28	24.02	0.35	0.32	0.36	FB	FB	FB

## Data Availability

The data presented in this study are available on request from the corresponding author.

## References

[1] Valdevit L., Vermaak N., Zok F.W., Evans A.G. (2008). A Materials Selection Protocol for Lightweight Actively Cooled Panels. J. Appl. Mech..

[2] Liu F., Jiang X., Wang X., Wang L. (2020). Machine learning-based design and optimization of curved beams for multistable structures and metamaterials. Extreme Mech. Lett..

[3] Abueidda D.W., Koric S., Sobh N. (2020). Topology optimization of 2D structures with nonlinearities using deep learning. Comput. Struct..

[4] Lv W., Li D., Dong L. (2020). Study on mechanical properties of a hierarchical octet-truss structure. Compos. Struct..

[5] Peng C., Tran P. (2020). Bioinspired functionally graded gyroid sandwich panel subjected to impulsive loadings. Compos. Part B Eng..

[6] Ashby M.F., Evans A.G., Fleck N.A., Gibson L.J., Hutchinson J.W., Wadley H.N.G. (2000). Metal Foams A Design Guide.

[7] Ashby M.F., Cebon D. (1993). Materials selection in mechanical design. Le J. De Phys. IV.

[8] Nguyen N.T., Siegmund T., Tsutsui W., Liao H., Chen W. (2016). Bi-objective optimal design of a damage-tolerant multifunctional battery system. Mater. Des..

[9] Attar P., Galos J., Best A., Mouritz A. (2020). Compression properties of multifunctional composite structures with embedded lithium-ion polymer batteries. Compos. Struct..

[10] Wadley H.N.G. (2005). Multifunctional periodic cellular metals. Philos. Trans. R. Soc. A Math. Phys. Eng. Sci..

[11] Lu T.J., Valdevit L., Evans A. (2005). Active cooling by metallic sandwich structures with periodic cores. Prog. Mater. Sci..

[12] Valdevit L., Pantano A., Stone H., Evans A. (2006). Optimal active cooling performance of metallic sandwich panels with prismatic cores. Int. J. Heat Mass Transf..

[13] Meng H., Galland M.A., Ichchou M., Bareille O., Xin F.X., Lu T.J. (2017). Small perforations in corrugated sandwich panel sig-nificantly enhance low frequency sound absorption and transmission loss. Compos. Struct..

[14] Tang Y., Ren S., Meng H., Xin F., Huang L., Chen T., Zhang C., Lu T. (2017). Hybrid acoustic metamaterial as super absorber for broadband low-frequency sound. Sci. Rep..

[15] Wadley H., Dharmasena K., O’Masta M., Wetzel J., O’Masta M.R. (2013). Impact response of aluminum corrugated core sandwich panels. Int. J. Impact Eng..

[16] Wadley H., O’Masta M., Dharmasena K., Compton B., Gamble E., Zok F. (2013). Effect of core topology on projectile penetration in hybrid aluminum/alumina sandwich structures. Int. J. Impact Eng..

[17] Ni C., Hou R., Xia H., Zhang Q., Wang W., Cheng Z., Lu T. (2015). Perforation resistance of corrugated metallic sandwich plates filled with reactive powder concrete: Experiment and simulation. Compos. Struct..

[18] Rathbun H., Zok F.W., Evans A. (2005). Strength optimization of metallic sandwich panels subject to bending. Int. J. Solids Struct..

[19] Cotea F., Deshpande V.S., Fleck N.A., Evans A.G. (2006). The compressive and shear responses of corrugated and diamond lattice materials. Int. J. Solids Struct..

[20] Zhao Z.-Y., Han B., Wang X., Zhang Q., Lu T.J. (2018). Out-of-plane compression of Ti-6Al-4V sandwich panels with corrugated channel cores. Mater. Des..

[21] Jiang W., Ma H., Yan L., Wang J., Han Y., Zheng L., Qu S. (2019). A microwave absorption/transmission integrated sandwich structure based on composite corrugation channel: Design, fabrication and experiment. Compos. Struct..

[22] Wang X., Zhao Z.-Y., Li L., Zhang Z.-J., Zhang Q.-C., Han B., Lu T.J. (2019). Free vibration behavior of Ti-6Al-4V sandwich beams with corrugated channel cores: Experiments and simulations. Thin-Walled Struct..

[23] Simonelli M., Tse Y.Y., Tuck C. (2014). Effect of the build orientation on the mechanical properties and fracture modes of SLM Ti–6Al–4V. Mater. Sci. Eng. A.

[24] Tancogne-Dejean T., Spierings A.B., Mohr D. (2016). Additively-manufactured metallic micro-lattice materials for high specific energy absorption under static and dynamic loading. Acta Mater..

[25] Bremen S., Meiners W., Diatlov A. (2012). Selective Laser Melting: A manufacturing technology for the future?. Laser Tech. J..

[26] Fousova M., Vojtech D., Kubasek J., Jablonska E., Fojt J. (2017). Promising charcteristics of gradient porosity TI-6Al-4V alloy pre-pared by SLM process. J. Mech. Behav. Bioed..

[27] Gautam R., Idapalapati S., Feih S. (2018). Printing and characterisation of Kagome lattice structures by fused deposition modelling. Mater. Des..

[28] Vayssette B., Saintier N., Brugger C., El May M. (2020). Surface roughness effect of SLM and EBM Ti-6Al-4V on multiaxial high cycle fatigue. Theor. Appl. Fract. Mech..

[29] (2009). ISO 6892-1:2009 Metallic materials—Tensile Testing. In Part 1: Method of Test at Room Temperature.

[30] Allen H.G. (1969). Analysis and Design of Structural Sandwich Panels.

[31] Roylance D. (2000). Beam Displacement. https://ocw.mit.edu/courses/materials-science-and-engineering/3-11-mechanics-of-materials-fall-1999/modules/MIT3_11F99_bdisp.pdf.

[32] Guo Y.L., Tong J.Z., Jiang Z.X. (2015). Design fundamentals and application of corrugated-web steel structures.

[33] Wilbert A., Jang W.-Y., Kyriakides S., Floccari J. (2011). Buckling and progressive crushing of laterally loaded honeycomb. Int. J. Solids Struct..

[34] Hohe J., Becker W. (2002). Effective stress-strain relations for two-dimensional cellular sandwich cores: Homogenization, material models, and properties. Appl. Mech. Rev..

[35] Yan L., Han B., Yu B., Chen C., Zhang Q.-C., Lu T. (2014). Three-point bending of sandwich beams with aluminum foam-filled corrugated cores. Mater. Des..

[36] Wei X.Y., Wu Q.Q., Gao Y., Xiong J. (2020). Bending characteristics of all-composite hexagon honeycomb sandwich beams: Experimental tests and a three-dimensional failure mechanism map. Mech. Mater..

